# The influence of Erythropoietin on platelet activation, thrombin generation and FVII/active FVII in patients with AMI

**DOI:** 10.1186/1477-9560-12-18

**Published:** 2014-08-28

**Authors:** Gabriele Demetz, Magdalena Laux, Armin Scherhag, Tiny Hoekstra, Marit M Suttorp, Friedo Dekker, Mark Roest, Mira Marcus-Kalish, Moshe Mittelman, Ilka Ott

**Affiliations:** 1 Deutsches Herzzentrum der Technischen Universität München, Lazarettstr. 36, München, 80636, Germany; 2 Pharmaceutical Division, F. Hoffmann–La Roche, Basel, Switzerland; 3 Department of Clinical Epidemiology, Leiden University Medical Center, Leiden, The Netherlands; 4 Department of Clinical Chemistry and Haematology, University Medical Center, Utrecht, The Netherlands; 5 Interdisciplinary Center for Technology Analysis & Forecasting (ICTAF), Tel Aviv University, Tel Aviv, 69978, Israel; 6 Department of Medicine A, Tel Aviv Sourasky Medical Center, Tel Aviv, Israel

**Keywords:** Platelet activation, Erythropoietin, AMI, PCI

## Abstract

**Background:**

Erythropoietin (Epo) has been shown to improve myocardial function in models of experimental myocardial infarction, but has also been associated with a rise in thromboembolic events. Thus, the aim of this study was to investigate the influence of Epo on platelet activation and coagulation in patients with acute myocardial infarction (AMI).

**Methods:**

The study was designed as a substudy of the randomised, double-blind, placebo controlled REVIVAL-3 (*REgeneration of VItal Myocardium in ST-Segment EleVation MyocardiAL Infarction by Erythropoietin)* study that investigated the effects of recombinant human Epo in AMI. Serial venous blood samples were collected before and after study medication. Circulating prothrombin fragment F1 + 2, FVII, active FVII, beta thromboglobulin (TG) and P-Selectin were measured before and 60 hours after randomization by immunoassay (n = 94). In a randomly selected subgroup platelet aggregation was measured using whole blood aggregometry (Multiplate Analyzer, n = 45).

**Results:**

After 5 days an increase in FVII was observed after Epo as compared to placebo (P = 0.02), yet active FVII and prothrombin fragment F1 + 2 remained unchanged. Moreover, no statistically significant differences in circulating TG or P-selectin were observed between the groups. As an expected response to peri-interventional therapy with clopidogrel and aspirin, platelet aggregation after stimulation with ADP, TRAP, ASPI or collagen decreased 12 hours and 2 days after PCI. However, no difference between the Epo and the placebo group was observed.

**Conclusion:**

After treatment with Epo in patients with AMI a slight increase in circulating FVII after Epo was not associated with an increase in active FVII, prothrombin fragment F1 + 2, TG or P-selectin. Moreover, platelet aggregation was not altered after treatment with Epo as compared to placebo.

**Trial registration:**

ClinicalTrials.gov Identifier: NCT01761435

## Introduction

Erythropoietin (Epo) is a hypoxia-induced hormone produced in the kidney that stimulates hematopoiesis in the bone marrow. As a therapeutic agent Epo became widely used in treating various types of anaemia including anaemia of end-stage renal disease, cancer-related anaemia on chemotherapy as well as anaemia associated with hematological neoplasms such as multiple myeloma and myelodysplatic syndromes.

Recent studies have also shown important non-hematopoietic effects of Epo. Functional Epo receptors are not only expressed on erythroid precursors, but also on megakaryocytes, vascular smooth muscle cells [1], endothelial cells [2], skeletal myoblasts [3], neurons [4], nephrons [5] and cardiac myocytes [6,7]. Numerous in vitro and in vivo studies have shown a protective role of human recombinant Epo during ischemia and reperfusion in the heart with a reduction in infarct size and apoptosis [8-11]. In the long term Epo may promote ischemia-induced neovascularization either by stimulating endothelial cells in situ or by mobilizing endothelial progenitor cells from the bone marrow [12,13].

High levels of endogenous erythropoietin in patients with acute myocardial infarction who underwent primary PCI were found to be associated with smaller infarct sizes [14]. First clinical studies showed the safety and feasibility of Epo or the long-acting Epo analogue darbepoetin-α administration in patients with acute MI and stroke [15,16]. However, Epo failed to fulfill the expectations of improving ischemia reperfusion injury that were raised in numerous experimental studies since no changes in myocardial function or infarct size were observed in five clinical trials [17-21]. Instead, an increase in adverse events in two of these trials [17,18] was observed.

Large clinical trials [22-24] in patients with chronic kidney disease showed an increase of thromboembolic events due to long-term Epo treatment, and short-term Epo treatment increased platelet count as well as platelet activation in animal models [25-28] and also in healthy human volunteers [29]. These findings may account for the unfavorable results of the clinical trials. We therefore sought to investigate the effect of Epo on platelet reactivity and coagulation markers prothrombin F1 + 2 and FVII in patients given Epo in acute myocardial infarction.

## Materials and methods

### Patients

One-hundred and thirty eight patients were recruited in the setting of The Regenerate Vital Myocardium by Vigorous Activation of Bone Marrow Stem Cells (REVIVAL-3) study [18] (ClinicalTrials.gov NCT00390832), a prospective, randomized and double-blind trial. The purpose of this study was to determine the value of Epo in patients with acute ST-elevation myocardial infarction in terms of improvement of left ventricular ejection fraction or reduction infarct size, both measured by magnetic resonance imaging. The study protocol was approved by the institutional ethics committee responsible for both participating centers, and all patients gave written informed consent for participation in the study. All patients received 600 mg of clopidogrel orally, 500 mg aspirin, and unfractionated heparin with or without abciximab intravenously. The study drug was given at 3 distinct time points: immediately after successful PCI in the catheterization laboratory and at 24 hours and 48 hours after random assignment. Each time, patients received either 3.33 × 10^4^ IU of recombinant human epoetin-β (NeoRecormon; F. Hoffmann-La Roche, Basel, Switzerland) or a matching placebo intravenously for 30 minutes. Post interventional antithrombotic therapy consisted of clopidogrel 75 mg twice a day for 3 days followed by 75 mg/d for at least 6 months. Aspirin 100 mg twice a day was recommended indefinitely. Other cardiac medications were prescribed at the discretion of the treating physician. Venous blood samples were collected before, 12 hours, 2, 3 and 5 days after treatment and citrate plasma was stored at −80°C. Of 94 randomly selected patients plasma samples were available and of 45 patients whole blood aggregometry was performed since the Multiplate Analyzer was not available at the beginning of the study.

### Immunoassays

To investigate the effect of the Epo treatment on coagulation and platelet activation markers plasma samples before and after completion of Epo therapy at day 3 were analyzed. Concentrations of prothrombin fragment F1 + 2, reflecting thrombin generation as a potent initiator of thrombocyte activation, were determined by immunoassay (Enzygnost F1 + 2 micro Behring Diagnostica). Detection limits were 0.04 nmol/L and intra-assay variabilities for the lower assay range were < 10%. At the University Medical Center Utrecht (Utrecht, The Netherlands), plasma levels of soluble platelet activation markers β-TG and P-selectin (R&D Systems Europe) were measured by semi-automated enzyme-linked immunosorbent assay (ELISA) on a TECAN Freedom Evo robot (Tecan, Mannedorf, Switzerland) as described previously with some minor modifications [30]. Minor modifications included: capture antibodies were coated overnight at 4°C; plasma samples were diluted 1/80 for measurement of β-TG and 1/10 for P-selectin; after incubation with biotinylated detection antibodies, streptavidin-poly-HRP were added. After incubation and washing, SuperSignal ELISA Pico chemiluminescent substrate was added and luminescence was measured (emission 470 nm) after 15 min incubation, with a SpectraMax L microplate reader from Molecular Devices Inc. (Menlo Park, CA, USA). The fluorogenic assay for FVII and FVIIa was modified as described [31]. Some of the levels were out of the detection range. We decided to fill in the highest measured value or the lowest measured value divided by 2.

### Aggregometry

Platelet aggregation was measured before, 12 hours, 2, 3 and 5 days after randomization immediately after collection, using whole blood aggregometry (Multiplate Analyzer, Verum Diagnostica) as described before [32], after stimulation with ADP, TRAP, ASPI or collagen. These time points were chosen to investigate effects of antiplatelet therapy.

### Other methods

Haemoglobin, haematocrit and platelet count were determined by automated routine analysis in the clinical chemistry laboratory.

### Statistical analysis

Changes between baseline and the follow-up timepoint were compared between groups using t-test. Changes over time were analyzed using repeated measures analysis of variance. P < 0.05 in the 2-tailed test was regarded as significant. Results are expressed as means ± SEM.

## Results

Baseline characteristics of the study patients showed minor differences in smoking and familial predisposition (Table 1).

**Table 1:** Baseline characteristics of the platelet study patients

**Characteritics**	**Epoetin-β (n = 23)**	**Placebo (n = 22)**
**Age (years)**	61.9 ± 2.5	61.3 ± 13.9
**Women**	6 (26)	7 (32)
**Men**	17 (74)	15 (68)
**Diabetes**	4 (17)	3 (14)
**Current smoker**	7 (30)	11 (50)
**Arterial hypertension**	14 (61)	13 (59)
**Hypercholesterinemia**	11 (48)	13 (59)
**Fam. Predisposition**	10 (43)	4 (18)

Data are presented as n (%) or mean ± SEM.

### FVIIa and thrombin generation

**Figure 1:**
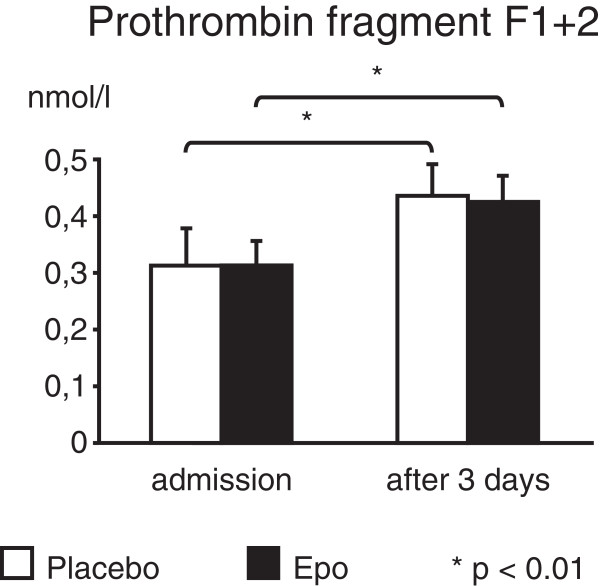
**Epo does not alter thrombin generation.** Prothrombin fragment F1 + 2 of patients treated with Epo or placebo on admission and after 60 hours. Values are expressed as mean + SEM. *p < 0.01 compared to the values on admission.

Thrombin generation in vivo, assessed by concentrations of prothrombin fragment F1 + 2, increased significantly after 3 days (p < 0.01, Figure 1). This effect was noticed both in the Epo group and the placebo group without any significant difference between the two groups. Measurement of FVII showed elevated total FVII after Epo as compared to the placebo group, yet active FVII remained similar between the groups (Table 2).

**Table 2:** Change in circulating platelet activation markers and FVII after Epo and placebo

**Marker**	**Before Epo**	**3 d after Epo**	**Before plac.**	**3 d after plac.**	**P-value**
**BetaTG (IU/ml)**	37.8 ± 4.4	47.7 ± 5.3	45.6 ± 6.1	43.5 ± 5.9	0.21
**P-selectin (ng/ml)**	140.0 ± 14.0	149.4 ± 15.0	153.9 ± 17.0	130.0 ± 13.2	0.19
**FVII (ng/ml)**	269.0 ± 36.1	291 ± 44.2	304.4 ± 53.9	229.3 ± 36.1	0.04
**Active FVII (ng/ml)**	17.9 ± 2.5	25.3 ± 3.2	20.6 ± 5.2	29.6 ± 6.4	0.8

Data are presented as mean ± SEM, P-values refers to changes between the intervention groups.

### Blood count

**Figure 2:**
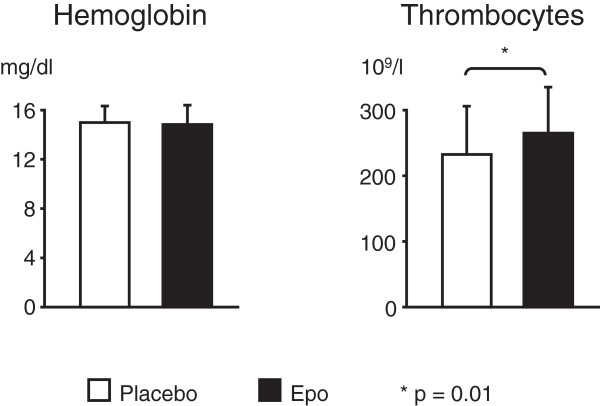
**Epo increases platelet count.** Haemoglobin and thrombocyte counts of patients treated with Epo or placebo after 5 days. Values are expressed as mean + SEM. *p = 0.01 in patients receiving Epo compared to those given placebo.

On admission haemoglobin (Epo 14.8 ± 0.3 mg/dl, placebo 14,3 ± 0.2 mg/dl), haematocrit (Epo 43 ± 0.5%, placebo 42 ± 0.5%)and platelet count (Epo 215 ± 56 × 10^9^/l, placebo 224 ± 71 × 10^9^/l) were comparable in both groups (n = 45). Similarly haemoglobin, haematocrit and platelet count were comparable in the subgroup with the platelet activation study (n = 45). After 5 days, the maximal platelet count was elevated in the Epo group (265 ± 70 × 10^9^/l) as compared to the placebo group (232 ± 74 × 10^9^/l, p = 0.011, Figure 2, n = 94). In contrast, haemoglobin levels did not differ between the two groups after 5 days (Epo 14.8 ± 1.6 versus placebo 15 ± 1.3 mg/dl, p = 0.593, Figure 2).

### Platelet reactivity

**Figure 3:**
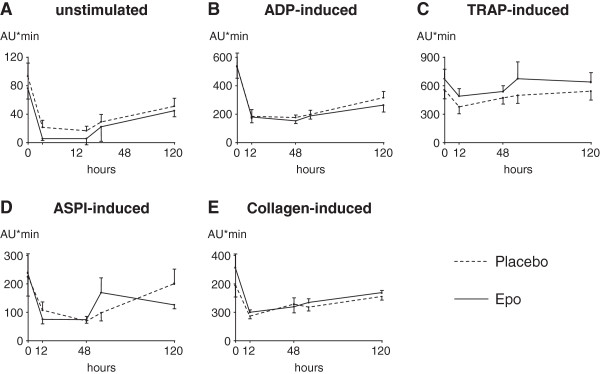
**Epo does not alter platelet aggregation.** Maximal platelet aggregation of patients treated with Epo or placebo on admission, after 12, 48 and 120 hours. Shown is the AUC of unstimulated platelets **(A)** and after stimulation with ADP **(B)**, TRAP **(C)**, ASPI **(D)** or collagen **(E)**. Values are expressed as mean + SEM.

After PCI, platelet aggregation decreased significantly as expected in response to periinterventional therapy with clopidogrel and aspirin (Figure 3A-E). This effect was most distinctive after 12 (TRAP- and collagen-induced, Figure 2C and E) hours and2 days (unstimulated cells and after stimulation with ADP and ASPI, Figure 2A,B and D), respectively. However, there was no significant difference between the Epo and the placebo group. After 3 and 5 days, platelet aggregation increased gradually but did not yet reach initial levels except for TRAP-stimulated platelets. Similarly, no statistically significant differences were observed in circulating platelet activation markers TG and P-selectin before and after 3 days in the Epo group as compared to the placebo group (Table 2).

## Discussion

Major findings of our study are as follows: (1) Although total FVII was elevated after treatment with Epo, no difference in the increase in active FVII and thrombin generation was observed between the two groups. (2) After treatment with Epo in patients with AMI and successful primary percutaneous coronary intervention the number of circulating platelets increased. (3) However, platelet activation was not altered after Epo as compared to the patients receiving placebo.

Platelets play a central role in the haemostatic process, including recognizing the site of injury, recruiting additional platelets by intercellular signaling, adhering to one another, and interacting with the coagulation cascade to form a hemostatic plug. Acute coronary syndromes are associated with inappropriate platelet activation which is crucial in the pathogenesis of thrombotic complications after percutaneous coronary interventions [33]. Moreover, platelet count is decreased in acute coronary syndrome as compared to stable angina [34]. Residual platelet reactivity is an independent predictor of myocardial injury in patients with acute myocardial infarction [35]. Experimental studies suggest an increase in platelet activation after short-term, high-dose Epo treatment [36] which may be deleterious for patients with acute myocardial infarction. The observation that Epo treatment increases platelet count reflects stimulation of megakaryocytopoiesis that have been observed in mice treated with high-dose, short-term Epo [37] yet this was not associated with increased platelet activation in patients with AMI receiving Epo.

In a non-acute coronary setting, in patients with long-term Epo treatment an increase of haematocrit is associated with increased rates of thromboembolic events [22-24]. Possible mechanisms therefore may be augmented platelet generation and release as well as increased platelet reactivity. On the other hand, non-hematopoietic mechanisms of Epo may contribute to favorable effects in acute myocardial infarction. A functional erythropoietin receptor was found in the cardiovascular system including endothelial cells [2] and cardiomyocytes [38], and several in vitro and in vivo studies have shown a protective role of human recombinant Epo during ischemia and reperfusion in the heart with a reduction in infarct size and apoptosis [8-11]. Despite this promising experimental data, no clinical benefit of Epo in acute myocardial infarction was found in several clinical trials [17-21]. In contrast, two of these studies showed a trend to increased adverse events [17,18]. As platelet activation is of particular importance when considering complications in acute myocardial infarction, the impact of Epo on platelets must be taken into account. Short term treatment with Epo has been associated with increased platelet count and activation in animal models [25-28] as well as in healthy human volunteers [29]. Accordingly, in our study we found an increased platelet count in patients receiving short-term, high-dose Epo in acute myocardial infarction. Yet since these changes are small they have no clinically relevance. Moreover, we did not observe higher platelet reactivity in patients treated with Epo. To examine this more thoroughly, we analyzed platelet aggregation with four different stimulants, but all stimulants as well as unstimulated platelets showed comparable results in the time course. This may be due to common peri-interventional antithrombotic therapy with aspirin and clopidogrel, as platelet aggregation declined significantly in the first 48 hours after PCI. Thus, a possible small difference between the two groups may be overridden. Similarly we did not find differences in the circulating platelet activation markers betaTG and P-selectin.

Although we found an increase in total FVII after treatment in the Epo group as compared to the placebo group active FVII was comparable in both groups. Thrombin generation increased after AMI and primary PCI as was described before [39], reflecting a general hypercoagulability as well as systemic inflammatory reactions, but we did not observe any difference between F1 + 2 concentrations in the Epo and placebo groups. Thus, activation of coagulation was comparable in both groups.

Experimental studies suggesting a protective role of Epo used dosages of 350 to 5000 IU/kg which corresponds to up to 350 000 IU in humans. Although the applied cumulative dosage of 100 000 IU in this study has been among the highest doses tested in clinical trials [15,40] no changes in platelet or coagulation activation were found.

Investigating the possible effect of Epo on coagulation and platelet function had another important consideration. Over the last decade several publications have raise the concern that cancer patients on Epo had a shorter survival than non-Epo treated patients [41]. Although other papers failed to confirm these concerns [42] and meta-analyses were inconclusive [43], caution is recommended. Three mechanisms for the potential harmful effects of Epo on tumors have been proposed [43] 1. activation of Epo receptors, 2. induced angiogenesis or 3. thromboembolism due to increased haematocrit, platelet activation or enhanced coagulation. Clinical trials and basic studies continue to explore this topic. However, the current study showing that platelet and other coagulation system functions are not affected, contributes to a better understanding of the mechanisms and probably excluding the possibilities that platelets are involved.

Limitations of the study are the small sample size, analysis of only one platelet activation assay and limited methods to study blood coagulation.

In conclusion, in the setting of AMI with standard concomitant medical therapy with aspirin and clopidogrel, short-term Epo increases platelet count but does not alter platelet aggregation or coagulation. Thus in these patients the use of Epo is safe.

## Abbreviations

ADP: Adenosine diphosphate; ASPI: Arachidonic acid; AUC: Area under the curve; Epo: Erythropoietin; TRAP: Thrombin receptor activating peptide.

## Competing interests

The authors declare that they have no competing interests.

## Authors’ contributions

GD, IO, AS have made substantial contributions to conception and design, GD and ML to acquisition of data, IO, TH, MMS, MR, to analysis and interpretation of data; FD, MM, IO, GD have been involved in drafting the manuscript or revising it critically for important intellectual content. All authors read and approved the final manuscript.
